# Identification of novel candidate variants including *COL6A6* polymorphisms in early-onset atopic dermatitis using whole-exome sequencing

**DOI:** 10.1186/s12881-017-0368-9

**Published:** 2017-01-26

**Authors:** Won Il Heo, Kui Young Park, Taewon Jin, Mi-Kyung Lee, MinJeong Kim, Eung Ho Choi, Hae-Suk Kim, Jung Min Bae, Nam Ju Moon, Seong Jun Seo

**Affiliations:** 1 Department of Dermatology, Chung-Ang University Hospital, Heukseok-Ro 102, Dongjak-Gu, Seoul, South Korea; 2 Department of Laboratory Medicine, Chung-Ang University Hospital, Seoul, South Korea; 3 Department of Dermatology, Yonsei University Wonju College of Medicine, Wonju, South Korea; 4 TheragenEtex Bio Institute Inc., Suwon, South Korea; 5 Department of Dermatology, St. Vincent’s Hospital, College of Medicine, Catholic University of Korea, Suwon, South Korea; 6 Department of Ophthalmology, Chung-Ang University Hospital, Seoul, South Korea

**Keywords:** *COL6A6*, Atopic dermatitis, Sanger sequencing, Whole-exome sequencing

## Abstract

**Background:**

The prevalence of atopic dermatitis has increased over the last 10 years. Atopic dermatitis tends to run in families and commonly begins to manifest in childhood. The prevalence of atopic dermatitis is as high as 20% in children. Thus, early diagnosis and treatment of atopic dermatitis are important. Understanding its genetic basis is also needed to facilitate early detection.

**Methods:**

To identify family-specific candidate genetic variants associated with early-onset atopic dermatitis in Koreans, we carried out whole-exome sequencing of three separate families with this condition. Additional validation was performed in 112 AD patients and 61 controls using Sanger sequencing.

**Results:**

We focused on both common functional variants with a minor allele frequency higher than 1% and rare variants with a minor allele frequency less than 1%. The relevance of the respective variants was supported by a program that could predict whether the mutations resulted in damaged protein function. Fourteen overlapping genes were identified during exome sequencing. Three variants of the *COL6A6* gene appeared in all three families and were in close proximity to atopic dermatitis-related loci on chromosome 3q21. The homozygous frequency for the rs16830494 minor allele (AA) and the rs59021909 (TT) allele and the rs200963433 heterozygous (CT) frequency were all higher in AD cases compared to controls in a population-based case-control study.

**Conclusion:**

Identifying family-specific *COL6A6* polymorphisms and genetic variants of other candidate genes associated with AD using WES is a novel approach. Our study suggests that *COL6A6* variants may be risk factors for atopic dermatitis. This study provides a genetic basis for early-onset AD diagnosis in Korean patients and the development of new therapies.

**Trial registration:**

Trial registration number: IRB NO. C2008030 (133); Name of registry: The collection research of clinical data and patient blood to identify genetic and protein biomarker of atopic dermatitis; Date of registration: 09-July-2008.

Trial registration number: IRB NO. C2015258 (1716); Name of registry: The collection study of patient blood and clinical data for the development of the prognosis prediction and early diagnosis of atopic dermatitis; Date of registration: 15-jan-2016.

**Electronic supplementary material:**

The online version of this article (doi:10.1186/s12881-017-0368-9) contains supplementary material, which is available to authorized users.

## Background

Atopic dermatitis (AD) is a chronic, relapsing, inflammatory skin disorder characterized by eczematous lesions and dry, itchy skin. AD seems to be caused by a combination of hereditary and environmental factors. Although AD has features of a multigenic syndrome, it tends to run in families and commonly begins to manifest in childhood [1]. In a large cohort study of family history, AD was determined to be inherited in an autosomal dominant fashion [2]. There are strong genetic heritable components in many other common and complex diseases [3].

AD occurs at a rate as high as 20% in children [4]. Understanding the genetic background, early discovery, and best therapies for AD is important. Thus, identification of causal variants associated with a common complex trait like AD is needed for early detection.

Whole-exome sequencing (WES) is a technique that involves the sequencing of all protein-coding genes, known as the exome, which is comprised of about 3×10^7^ bases. Although the exome constitutes less than 2% of the human genome, mutations in the exome can have more severe consequences than do mutations in the other 98% of the human genome [5]. The purpose of WES is to identify variations by filtering big data collected from all protein-coding regions. This data includes disease-causing mutations inherited in a Mendelian pattern or disease-predisposing single-nucleotide polymorphisms (SNPs) found in both common and complex disorders [6]. To identify familial causative variants of early-onset AD, we recruited three pedigrees from families with a history of AD and severe clinical phenotypes. We then performed WES on all involved individuals. Alleles were compared to identify causal variants and subsequently validated in a population-based case-control study using Sanger sequencing.

A considerable number of rare and common variants were found, and 14 overlapping genes were detected in the three families. The common disease-common variant (CD-CV) hypothesis can best be tested in genome-wide association studies (GWASs). However, the hypothesis that individually gathered rare variants have severe effects arose from issues of missing heritability in the CD-CV hypothesis [7]. Common variants of common diseases were recently reported to be shared among different races [8]. The importance of common and rare variants is still uncertain [9]. Thus, we aimed to identify not only rare variants, but also common variants.

Linkage analysis is used to determine the rough positions of causal genes relative to known genetic makers. An AD linkage analysis found that chromosome region 1q21 contains an epidermal differentiation complex (EDC). The EDC includes various AD-related genes (e.g., loricrin, involucrin, filaggrin, and the S100 family) [10]. We used AD-related loci to confirm the association of the 14 overlapping genes with AD [11].

Here, we report the WES results from familes with early-onset AD, and we suggest the possibility of new variants, *COL6A6* polymorphisms, as novel candidate for the detection of early-onset AD.

## Methods

### Patients

Peripheral blood samples were obtained from three families with a history of AD. Each family consists of 2 affected and 2 unaffected individuals (Additional file 1: Figure S1). We attempted to eliminate environmental factors as much as possible by recruiting early-onset cases. This study was reviewed and approved by the Chung-Ang University Hospital Institutional Review Board. Each family member was diagnosed with AD by a dermatologist. All patients and children developed AD before 2 years of age and were selected based on high IgE level (>1000) and SCORAD score (>50). Additionally, 112 AD patients and 61 control subjects under 2 years 9 months old were enrolled to validate the association between the candidate variants and atopic dermatitis (Additional file 1: Table S1).

### Whole-exome sequencing

Genomic DNA was isolated from the peripheral blood of the members of the three families using a QIAamp DNA Mini Kit (Qiagen Inc, Valencia, CA, USA). The DNA quality and quantity were assessed with a Nanodrop spectrometer (Nanodrop Technologies, Wilmington, DE, USA) and a Qubit fluorometer (Life Technologies, Grand Island, NY, USA). WES was performed using SureSelect Human All Exon V4 + UTR 71 Mb (Agilent, CA, USA), following the manufacturer’s standard protocol. Genomic DNA was sheared using Covaris (Covaris, Woburn, MA, USA). A paired-end DNA sequencing library was prepared through shearing, end-repair, A-tailing, peak detection, PE adaptor ligation, and amplification. After the library was hybridized with bait sequences for 24 h, it was purified and amplified with an index barcode tag, and the library quality and quantity were determined. The exome library was sequenced with the 100-bp paired-end mode of the HiSeq SBS kit.

### Whole-exome sequencing processing and alignment

Sequence reads in FASTQ format were mapped to the human assembly UCSC hg19 using the Burrows-Wheeler Aligner (BWA, v0.7.7) [12] with “mem” and seed value parameters “-k 45” to create SAM files with correct mate pair information. The read group tag included the sample name. Picard (v1.92) was then used to convert the SAM files to compressed BAM files and then to sort the BAM files by chromosome coordinate. The Genome Analysis Toolkit (v2.3.9Lite) [13] was used to locally realign the BAM files at intervals corresponding to potential insertion/deletion (indel) alignment errors. Insertions and deletions were identified with Mutect [14] and a GATK Somatic Indel Detector, respectively. Single-nucleotide variants and indels were annotated using snpEff (v3.6c) [15] to classify variants as synonymous, non-synonymous, missense, frameshift point mutations, or frameshift indels.

### Annotation

Filter 1: SnpEff (http://snpeff.sourceforge.net/index.html) is a type of variant annotation and an effect prediction tool. It annotates and predicts the effects of variants, such as amino acid changes, on genes. Variants produce effects of different “types” (e.g., non-synonymous, stop-gained, insertion, deletion).

Filter 2: Impact prediction by SnpEff shows results of putative variants, making it easier to quickly categorize and prioritize variants (High: Splice_Site_Acceptor, Splice_Site_Donor, Start_Lost, Frame_Shift, Stop_Gained; Moderate: Non_Synonymous_coding, Codon_Change, Codon_Insertion and Deletion, etc.).

Filter 3: SIFT and Polyphen2 of dbNSFP

The SIFT score predicts whether an amino acid substitution affects protein function. SIFT prediction is based on the degree of amino acid conservation in aligned segments derived from closely related sequences, as collected through PSI-BLAST. The range was 0 to 1; substitutions with scores lower than 0.05 were predicted to be damaging (a lower score signified a greater detrimental effect), whereas substitutions with higher scores were predicted to be tolerable.

The Polyphen2 HDIV score was based on HumDiv, i.e., hdiv_prob. The score ranged from 0 to 1, and a higher score suggested a greater degree of predicted damage. A prediction of “probably damaging” corresponded to scores between 0.957 and 1, “possibly damaging” for scores ranging from 0.453 to 0.956, and “benign” for those between 0 and 0.452.

Filter 4: The PhyloP of dbNSFP detects sites under negative or positive selection while allowing for changes in evolution rate over the branches of the phylogenetic tree. A higher PhyloP score indicates a better conserved site (http://varianttools.sourceforge.net/Annotation/DbNSFP).

Filter 5: PhastCons measures the strength of purifying selection acting on a DNA sequence. A high PhastCons score (0.2) is strong evidence that a genomic region is functionally important.

Filter 6: The 1000 Genome allele frequency of dbNSFP selects variants with frequencies of less than 0.01 or those with unknown frequencies.

Filter 7: The in-house Korean database at the Theragen Etex Bio Institute selects variants with minor allele frequencies (MAFs, less than 0.02 or unknown).

### Sanger sequencing

Three SNPs were selected for Sanger validation. PCR amplification of all SNPs was performed at 95 °C for 10 min, followed by 35 cycles at 95 °C for 30 s, 55–58 °C for 30 s and 72 °C for 40 s, with a final extension at 72 °C for 1 min 30 s. The PCR mixtures (total volume 50 μL) contained 25 μL of 2X EF-Taq premix (SolGent, Seoul, South Korea), 2.5 μL of oligonucleotide primer (10 pmol/μL), 18 μL of distilled water, and 2 μL of template containing 20 ng genomic DNA. The PCR products underwent purification via a PCR purification kit (Favorgen, Pingtung, Taiwan) and were sequenced on an Applied Biosystems 3500 DNA sequencer (Foster City, CA, USA) according to the manufacturer’s instructions.

## Results

### Whole-exome sequencing in three families with atopic dermatitis

WES was performed on three families with AD. To limit our study to genetic factors, we recruited three pedigrees from families with severe clinical AD phenotypes and attempted to minimize environmental factors by selecting early-onset cases. The WES analysis showed that all affected individuals were heterozygous for the identified variants, while unaffected individuals were homozygous wild-type (Table 1).

**Table 1:**
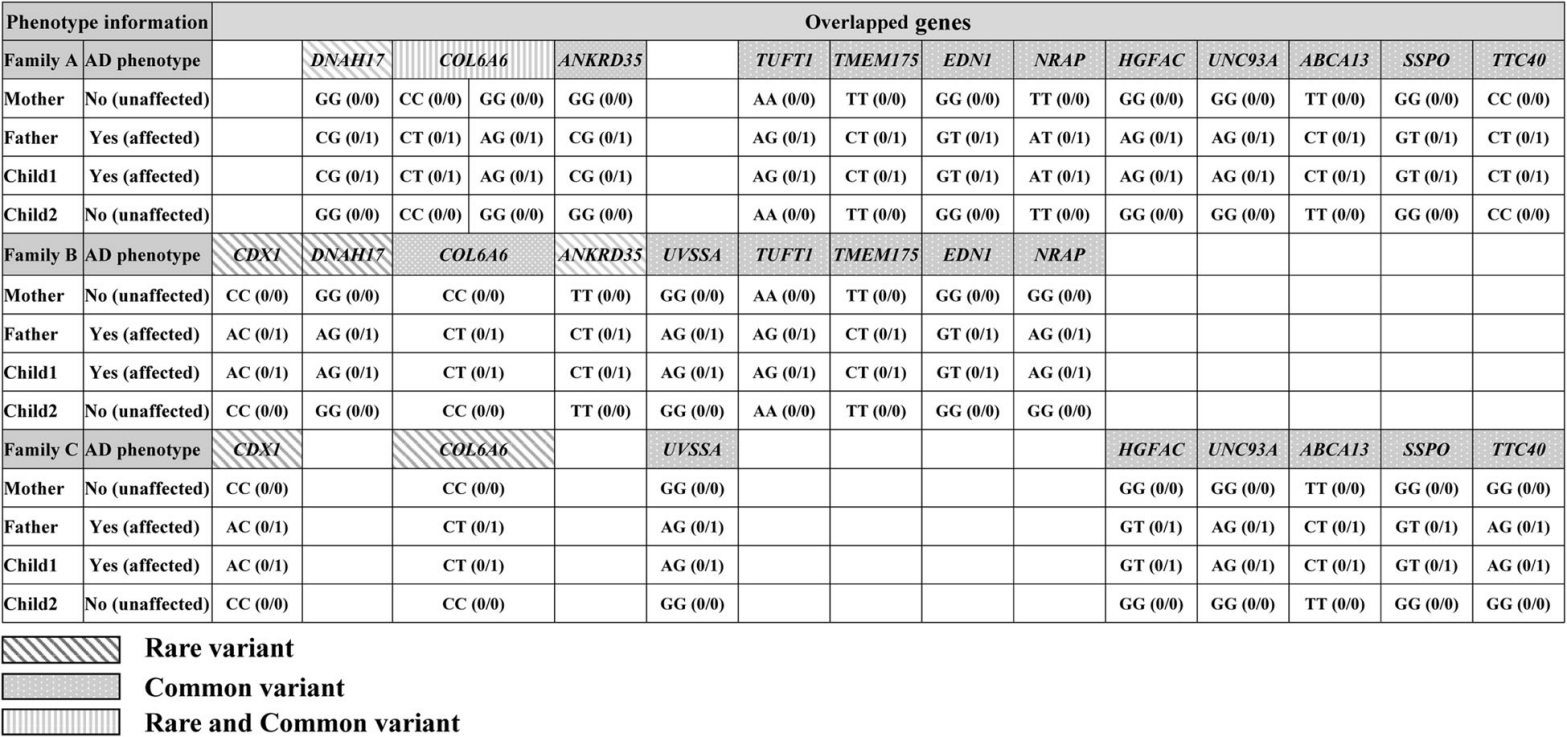
Genotypes of overlapping common and rare variants in three families

A large amount of WES data from each individual exome were filtered in a stepwise fashion to isolate candidate variants related to AD (Additional file 1: Figure S2). The variants collected from the three families were counted after each filtering process. We obtained an average of 176 common variants per family after filter 4 and an average of 48 rare variants following the 1000 Genome filter 5. An average of 44 rare variants were detected after the Korean filter (Table 2).

**Table 2:** Number of variants by filter in three families using a dominant genetic model

Filter system		Dominant model		
Filter step	Filter process	Family A	Family B	Family C
Raw	–	7523	3564	3227
Filter 1	EFFECT	2799	2489	2198
Filter 2	IMPACT	502	516	443
Filter 3	SIFT, PolyPhen2	278	258	231
Filter 4	phyloP	234	225	206
Filter 5	Phastcon	200	173	156
Filter 6	1000 genome	33	60	53
Filter 7	Korean	31	54	49

Amino acid changes and variant types (non-synonymous SNP, stop-gained, insertion, deletion) were determined using the variant annotation from the step 1 filter. A non-synonymous SNP is a single-nucleotide change that results in a codon for a different amino acid. Considerable numbers of non-synonymous SNPs were observed in the three families (data not shown).

The variants identified by filter 5 not only indicated common variants (MAF greater than 1%), but also possible functional variants predicted to impair protein function as well as sequences that are highly conserved among 100 vertebrate species.

To find more critical variants among the many filtered genes, we confirmed the overlapping genes of common variants from filter 5 in the three families (Table 1 and Additional file 1: Figure S3). There were 14 overlapping genes in filter 5, four of which reached filter 7 and could be called “rare variants.” Risk alleles were identified in AD patients by comparison with healthy controls (Table 1). The number of overlapping genes is also depicted as a Venn diagram (Additional file 1: Figure S3). Variants of *COL6A6* appeared in all three families (Tables 3, 4 and 5), and two *COL6A6* SNPs were detected in Family A (Table 3).

**Table 3:** Family-specific common and rare variants in Family A

	Common variant						Functional prediction program					
Gene	RS#	^a^Chr	^b^POS	^c^Amino acid change	Type	^d^SIFT	^e^Polyphen2	^f^PhyloP	^g^PhastCons	^h^Global	East Asian	^i^Korean
*COL6A6*	16830494	chr3	130,361,856	R1739Q	cSNP	0.04	0.272	1.703	0.995	0.12	0.19	0.188
*ANKRD35*	11579366	chr1	145,562,293	E661Q	cSNP	0.38	0.971	5.285	1	0.39	0.26	0.233
*TUFT1*	3828054	chr1	151,512,895	Q18R	cSNP	0.77	0.688	1.688	0.288	0.1	0.03	0.047
*TMEM175*	34311866	chr4	951,947	M393T	cSNP	0.01	0	1.299	0.999	0.12	0.12	0.155
*EDN1*	5370	chr6	12,296,255	K198N	cSNP	0.08	0.454	0.049	0.001	0.21	0.28	0.258
*NRAP*	2270182	chr10	115,392,919	N519I	cSNP	0.19	0.958	2.477	1	0.26	0.20	0.205
*HGFAC*	16844401	chr4	3,449,652	R509H	cSNP	0.22	0.943	2.662	0.593	0.07	0.10	0.115
*UNC93A*	2235197	chr6	167,709,702	W151	STOP GAINED	.	.	4.525	1	0.1	0.15	0.163
*ABCA13*	1771229	chr7	48,313,881	F1540L	cSNP	.	0.997	2.325	0.614	0.11	0.17	0.255
*SSPO*	1005603	chr7	149,516,881	S4028I	cSNP	.	.	.	.	0.2	0.19	0.181
*TTC40*	12781609	chr10	134,748,331	S264N	cSNP	.	.	.	.	0.37	0.36	0.412
Rare variant												
*DNAH17*	.	chr17	76,567,792	I204M	cSNP	0.11	.	1.926	0.992	.		.
*COL6A6*	200963433	chr3	130,289,976	R906C	cSNP	0	1	4.596	1	0.0014	0.01	0.017

^a^Chr = Chromosome

^b^Pos = Position

^c^Amino acid changes = single-letter codes for amino acids are presented, cSNP = single-nucleotide polymorphisms in coding regions

^d^SIFT, prediction scores for amino acid substitutions that affect protein function (damaging < 0.05, tolerance > 0.05; scores range from 0 to 1)

^e^Polyphen2, prediction of the possible impact of amino acid substitutions (0.957 < probably damaging < 1, 0.453 < possibly damaging < 0.956, 0 < benign < 0.452; scores range from 0 to 1)

^f^PhyloP, prediction of conserved sites across species; a higher score indicates a more conserved site (values > 0)

^g^PhastCons, predicts the possibility that a nucleotide belongs to a conserved element in the phylogenetic tree (Values > 0.2)

^h^Global frequency, variants with MAFs as low as 1% or with an unknown frequency (value < 0.01)

^i^Korean frequency, variants with MAFs as low as 2% or in unknown genes (values > 0.02)

**Table 4:** Family-specific common and rare variants in Family B

	Common variant						Functional prediction program					
Gene	RS#	Chr	POS	Amino acid change	Type	SIFT	Polyphen2	PhyloP	PhastCons	Global	East Asian	Korean
*COL6A6*	59021909	chr3	130,285,929	P556S	cSNP	0.11	0.999	2.136	0.997	0.09	0.08	0.119
*UVSSA*	2276904	chr4	1,349,029	R391H	cSNP	.	.	.	.	0.23	0.42	0.374
*TUFT1*	3828054	chr1	151,512,895	Q18R	cSNP	0.77	0.688	1.688	0.763	0.1	0.03	0.047
*TMEM175*	34311866	chr4	951,947	M393T	cSNP	0.01	0	1.299	0.563	0.12	0.12	0.155
*EDN1*	5370	chr6	12,296,255	K198N	cSNP	0.08	0.454	0.049	0.895	0.21	0.28	0.258
*NRAP*	868738	chr10	115,381,747	R884C	cSNP	0.01	0.986	4.833	1	0.24	0.17	0.198
Rare variant												
*CDX1*	370852694	chr5	149,546,819	A127E	cSNP	0.87	0.458	1.235	0.996	.	.	0.027
*DNAH17*	78098467	chr17	76,510,974	A1332V	cSNP	0.6	.	4.052	1	0.01	0.04	0.037
*ANKRD35*	146839643	chr1	145,560,094	C194R	cSNP	0.01	1	3.419	1	0.0037	0.01	0.016

**Table 5:** Family-specific common and rare variants in Family C

	Common variant						Functional prediction program					
Gene	#RS	Chr	POS	Amino acid change	Type	SIFT	Polyphen2	PhyloP	PhastCons	Global	East Asian	Korean
*UVSSA*	2276904	chr4	1,349,029	R391H	cSNP	.	.	.	.	0.23	0.42	0.374
*HGFAC*	3748034	chr4	3,446,091	A218S	cSNP	0.38	0.659	2	1	0.15	0.28	0.305
*UNC93A*	2235197	chr6	167,709,702	W151	STOP GAINED	.	.	4.525	1	0.1	0.15	0.163
*ABCA13*	17712299	chr7	48,313,881	F1540L	cSNP	.	0.997	2.325	0.85	0.11	0.17	0.255
*SSPO*	1005603	chr7	149,516,881	S4028I	cSNP	.	.	.	1	0.2	0.19	0.181
*TTC40*	.	chr10	134,679,632	T1596M	cSNP	.	.	.	.	.		.
Rare variant												
*COL6A6*	200963433	chr3	130,289,976	R906C	cSNP	0	1	4.596	1	0.0014	0.01	0.017
*CDX1*	370852694	chr5	149,546,819	A127E	cSNP	0.87	0.458	1.235	0.996	.	.	0.027

The genotypes of the 14 genes were further assessed in an exome analysis. Affected fathers and children were heterozygous, while unaffected mothers and children were homozygous wild type for all 14 candidate genes (Table 1).

The chromosome position, amino acid change, and functional prediction score for each of the 14 overlapping genes are presented for each family. The 14 candidate genes were deemed functionally interesting and supported by the SIFT scores (probability of being damaging/deleterious) and the results of the PhyloP analysis (highly conserved among 100 vertebrate species). The significantly low SIFT and high PhyloP and Phastcon scores of *COL6A6* found in all three families signify deleterious protein function. Furthermore, when the Korean filter was applied to *COL6A6*, all three families showed a genetic variant with an MAF range of 1.7–18%, as measured in 800 Koreans subjects (Tables 3, 4 and 5).

**Fig. 1:**
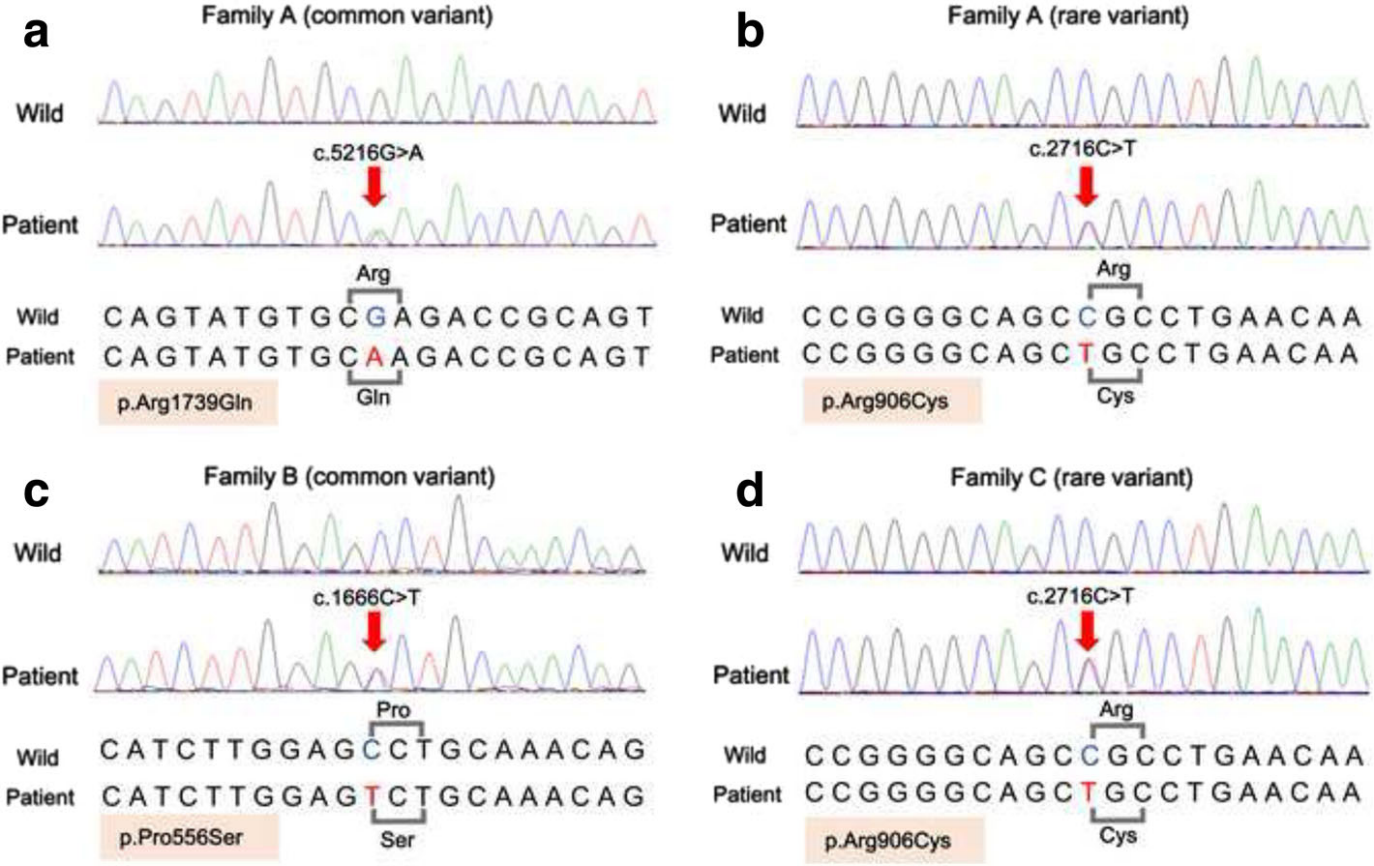
Single-nucleotide polymorphisms (SNPs) in coding regions of *COL6A6* in three families. The missense mutation was detected by Sanger sequencing analysis in each family (**a**-**d**)

To reduce errors from WES, *COL6A6* was analyzed by Sanger sequencing to detect SNPs in all three families. The variants we identified were in positions consistent with those of the WES results. In Family A, the missense mutations c.5216G > A, p.Arg1739Glu (common variant), and c.2716C > T, p.Arg906Cys (rare variant) were detected in the affected family members, but not in unaffected family members (Fig. 1a and b). In Family B, the missense mutation c.1666C > T, p.Pro555Ser (common variant) was only found in the affected members (Fig. 1c). In Family C, the missense mutation c.2716C > T, p.Arg906Cys (rare variant) was also only detected in the affected members (Fig. 1d).

Additionally, to investigate the possibility of *COL6A6* variants (rs16830494, rs59021909, and rs200963433) as candidate risk factors for AD, Sanger sequencing was also performed in a case-control study involving 112 patients with AD and 61 control subjects. The allele and genotype frequencies were counted (Table 6). Odds ratios (OR) and 95% confidence intervals (CI) were estimated for all risk factors (Additional file 1: Table S2). No significant associations were found in these three variants under either dominant or recessive models. However, a minor allele (A) of the common variant rs16830494 showed a tendency toward lower frequency. Homozygosity for the rs16830494 minor allele (AA) and for the rs59021909 (TT) allele were more frequent in AD cases compared to controls. The rs200963433 is a rare variant; the 3.5% heterozygous frequency (CT) of rs200963433 seen in the AD cases was double the 1.6% frequency observed in healthy controls (Table 6). The frequency of rs200963433 in Sanger sequencing was also compared with the frequency of 1000 global, 286 East Asian, and 800 Korean subjects. The 1.7% MAF of rs200963433 in the 800 Koreans surveyed nearly matched that of the 61 health controls in Sanger sequencing, and the MAF was elevated in AD cases (Tables 3 and 6).

**Table 6:** Allele and genotype frequencies of *COL6A6* polymorphisms in 112 Korean AD patients and 61 controls

SNP			Controls, n (%)	AD, n (%)
rs16830494	Allele	G	99 (81.1)	189 (84.4)
(c.5216G > A)		A	23 (18.9)	35 (15.6)
	Genotype	GG	39 (63.9)	79 (71.2)
		GA	21 (34.4)	29 (26.1)
		AA	1 (1.6)	3 (2.7)
rs59021909	Allele	C	108 (88.5)	199 (88.8)
(c.1666C > T)		T	14 (11.5)	25 (11.2)
	Genotype	CC	47 (77.0)	89 (79.5)
		CT	14 (23.0)	21 (18.8)
		TT	0 (0)	2 (1.8)
rs200963433	Allele	C	121 (99.2)	220 (98.2)
(c.2716C > T)		T	1 (0.8)	4 (1.8)
	Genotype	CC	60 (98.4)	108 (96.4)
		CT	1 (1.6)	4 (3.6)
	.	TT	0 (0)	0 (0)

We compared the genetic loci between AD candidate variants identified via WES and in AD-linked chromosomal regions. In previous studies, AD-related chromosome loci were detected using AD linkage analysis. The *CDX1*, *ANKRD35*, and *TUFT1* genes were present at positions 5q31-33 and 1q21. *COL6A6* was in close proximity to the 3q21 locus, which is known to be linked to AD (Table 7).

**Table 7:** Five candidate genes within AD susceptibility loci identified through genetic linkage analysis

Gene	Chr	POS	Locus of AD-linkage
*COL6A6*	3	130.2–4 Mb^a^	3q21 (chr3 122.2 Mb–129.5 Mb) [11]
*CDX1*	5	149.5 Mb	5q31-33 (Chr5 131.2 Mb–160.5 Mb) [29]
*ANKRD35*	1	145.5 Mb	1q21 (Chr1 143.2 Mb–155.1 Mb) [10]
*TUFT1*	1	151.5 Mb	1q21 (Chr1 143.2 Mb–155.1 Mb)
*FLG*	1	152.2 Mb	1q21 (Chr1 143.2 Mb–155.1 Mb)

^a^Mb = mega base pairs = 1,000,000 bp

We also detected SNPs in filaggrin (*FLG*) and *FLG2* in these three families. *FLG* polymorphisms at the 1q21 locus were observed in three families, respectively (Additional file 1: Table S3).

## Discussion

Early-onset AD is a phenotype that may be associated with a higher risk of multiple allergies and asthma [16]. The identification of specific genes predictive of early-onset AD may lead to AD prevention and better management.

To identify familial candidate genes related to early-onset AD, we recruited three families with this phenotype. A family-based design has the advantages of being cost-effective and the ability to discover rare variants not detectable in a population study [17]. De novo gene mutations capable of influencing AD susceptibility can also be identified in affected individuals by comparison with unaffected individuals in a family.

Family-specific candidate AD genes were detected using WES. Considerable numbers of common and rare variants were identified in each of the three families. To identify highly critical genes for AD, we confirmed 14 overlapping genes in these families. Variants of the overlapping genes were predicted to be deleterious through functional prediction algorithms. The results of previous AD association studies and the functions of candidate genes were also examined.

Common and rare variants of *COL6A6* were found in all three families. The *COL6A6* gene encodes collagen type VI alpha 6, the α6-chain of an extracellular matrix protein that is widely expressed in human skin and maintains skeletal muscle and skin integrity [18]. *COL6A6* is in close proximity to 3q21. In previous studies, AD-associated loci were confirmed by whole-genome linkage scans. The 3q21 locus has been identified as an AD susceptibility region [11]. Another genome-wide linkage study found highly significant evidence of a linkage to chromosome 3q21. Moreover, significant evidence has linked this locus with allergic sensitization presumably by paternal imprinting, further supporting the presence of an atopy-related gene in this region [19]. *CD80* and *CD86* are major candidate genes located on 3q21 that are expressed by antigen-presenting cells and are essential to T cell activation [20]. *COL6A5*, the other collagen alpha-chain, is also linked to AD. A lack of *COL6A5* expression affects epidermal integrity and function [21]. Early onset-AD has a prevalence of 15–20% in industrialized countries [4]. The MAF (1.7–18%) of *COL6A6* variants in a large sample of the Korean population was similar to the incidence rate of early-onset AD. Our findings suggest that variants of *COL6A6* may be novel candidates for early-onset AD in Koreans.

No significant association with AD was identified among the three *COL6A6* variants per the Odds ratios used in the case-control study. However, a high frequency of homo alt was detected in rs16830494 and rs59021909 in AD cases relative to controls. It was difficult to obtain *p*-values for rare variants using Odds ratio, a common limitation [22]. The similar frequencies of rs200963433 in both the 800 Korean population and the 61 healthy controls demonstrate the credibility of the data despite the small study size.

A rare variant of the caudal type homeobox1 (*CDX1*) gene was detected in two families. *CDX1* is located in a candidate AD-linkage region, 5q31-33 [23]. The function of the *CDX1* gene is to inhibit β-catenin/T-cell factor transcriptional activity. β-catenin regulates cell-cell adhesion, and Wnt/β-catenin signaling is associated with skin development [24, 25]. A common variant of the ultraviolet (UV)-stimulated scaffold protein A (UVSSA) gene was also identified. The function of the *UVSSA* gene is to repair DNA damaged by UV rays. The function of any other genes assumed the association of AD was not found.

Missense mutations of *FLG* and *FLG2* were detected in three families, respectively. The effects of the FLG polymorphism on AD are not well characterized. However, Seon-Young et al. recently reported that the *FLG* (rs11584340) polymorphism is associated with a higher AD risk in the Korean population, and that it affects free fatty acids in serum of AD patients [26]. Loss‐of‐function mutations involving *FLG* strongly predispose the carrier to early-onset AD, but not to late-onset AD [27]. This study suggests that genetic screening is crucial for identifying risk variants of early-onset AD.

Individually gathered rare variants have severe effects and play important roles in complex human disorders [28]. Thus, our data will help to expand genetic studies of AD.

## Conclusions

Identifying family-specific *COL6A6* polymorphisms and genetic variants of other candidate genes associated with AD using WES is a novel approach. Our study suggests that *COL6A6* variants may constitute candidate risk factors for AD development, as identified via family-based WES and a non-familial case-control study of 173 subjects. This study provides a genetic basis for early-onset AD diagnosis in Korean patients and the development of new therapies.

## Additional file

Additional file 1: Table S1. Clinical characteristics of the subjects. **Table S2.** Odds ratios (ORs) and 95% confidence intervals (CIs) for atopic dermatitis associated with COL6A6 polymorphisms in Korean patients. **Table S3.** Fliaggrin variants detected in three families. **Figure S1.** Pedigree diagrams of three families with atopic dermatitis. **Figure S2.** Flow chart of the process for filtering variants. **Figure S3.** A Venn diagram showing the number of overlapping genes. (PDF 258 kb)

## Abbreviations

AD: Atopic dermatitis
CD-CV: Common disease-common variant
EDC: Epidermal differentiation complex
GWASs: Genome-wide association studies
MAF: Minor allele frequency
SNPs: Single-nucleotide polymorphisms
WES: Whole-exome sequencing
