# Supplementary material for: Identification of novel candidate variants including COL6A6 polymorphisms in early-onset atopic dermatitis using whole-exome sequencing
Source: BMC Med Genet. 2017 Jan 26;18:8. doi: 10.1186/s12881-017-0368-9 (PMC5270287; doi:10.1186/s12881-017-0368-9)

Supplementary methods

Statistical Analysis

Using logistic regression analysis, crude odds ratios (ORs), and 95% confidence intervals (CIs), associations between atopic dermatitis and the SNPs under investigation were calculated. Associations between *COL6A6* polymorphisms and AD were assessed using Fisher's exact test, as this test is most commonly used for this type of case-control study.

Supplementary data

Table S1. Clinical characteristics of the subjects

	**Healthy controls**	**Early onset-AD**
Total, *n*	61	112
Age, years, mean±SD	0.9 ± 0.7	1.8 ± 0.8
Male %	54.1%	63.4%
Total IgE, KU/L	-	159.5 ± 294.6
Eosinophils, mm^3	-	382.0 ± 422.9

Table S2. Odds ratios (ORs) and 95% confidence intervals (CIs) for atopic dermatitis associated with *COL6A6* polymorphisms in Korean patients.

SNP	Allele				Genotype (Dominant)				Genotype (Recessive)			
	Minor allele	Major allele	OR (95% CI)	*p*-value	Case	Control	OR (95% CI)	*p*-value	Case	Control	OR (95% CI)	*p*-value
**rs16830494**	A	G	0.7971 (0.4465 to 1.4231)	0.4431	AA+GA	GG	0.7181 (0.3694 to 1.3958)	0.3288	AA	GA+GG	1.6667 (0.1696 to 16.3780)	0.6613
**rs59021909**	T	C	0.9691(0.4837 to 1.9417)	0.9295	TT+TC	CC	0.8676 (0.4088 to 1.8413)	0.7114	TT	TC+CC	2.7828 (0.1315 to 58.9004)	0.5111
**rs200963433**	T	C	2.2000 (0.2431 to 19.9057)	0.4829	TT+TC	CC	2.2222 (0.2428 to 20.3374)	0.4796	TT	TC+CC	0.5020 (0.0098 to 25.6072)	0.7312

Table S3. Fliaggrin variants detected in three families

**AD-associated variants**							**Functional prediction program**						
**Gene**	**#RS**	**Chr**	**POS**	**Amino acid change**	**Type**	**Family**	**SIFT**	**Polyphen2**	**PhyloP**	**PhastCons**	**Global**	**East Asian**	**Korean**
FLG	.	chr1	152,277,578	A3262T	cSNP	A	1	0.995	0.628	0.009	.	.	.
FLG	80088153	chr1	152,276,803	Q3520P	cSNP	B	0.32	0.996	-0.086	.	0.01	0.05	.
FLG2	2282302	chr1	152,329,369	C298S	cSNP	C	0.97	0.965	-0.065	0.999	0.17	0.21	0.218

Figure S1. Pedigree diagrams of three families with atopic dermatitis. Squares indicate males, and circles indicate females. Solid symbols represent affected individuals with AD. Unshaded symbols represent unaffected individuals.

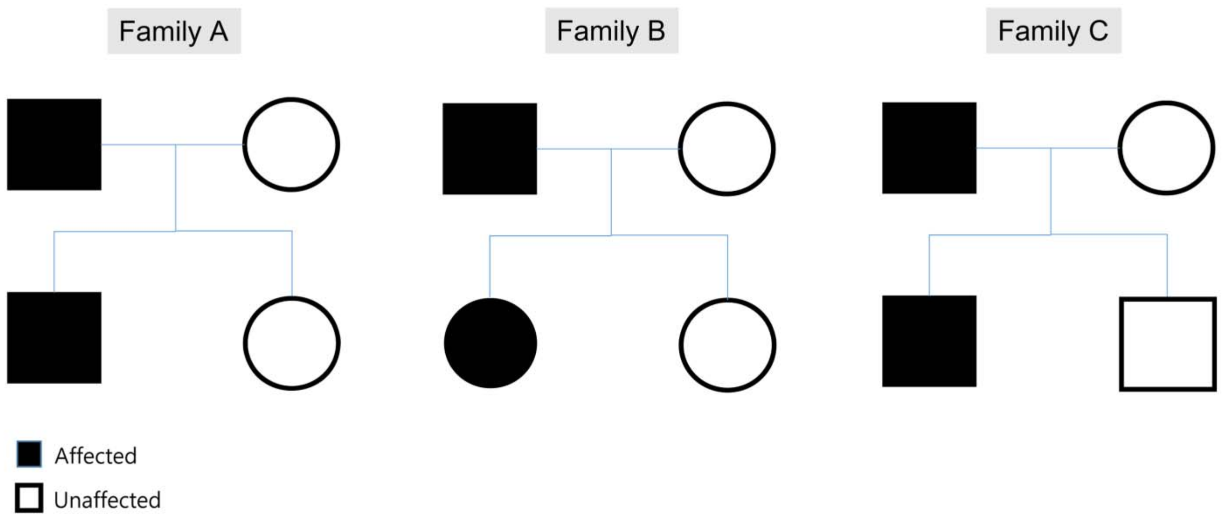

Figure S2. Flow chart of the process for filtering variants.

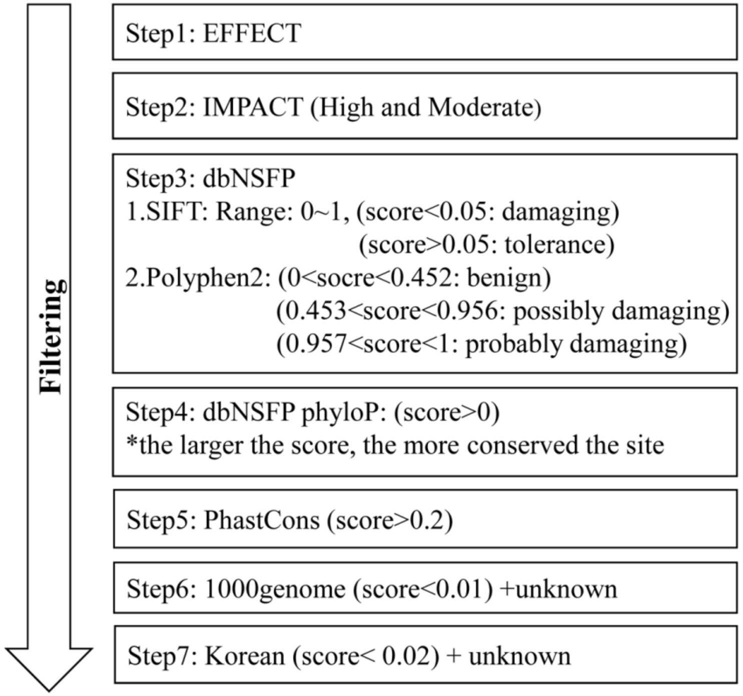

Figure S3. A Venn diagram showing the number of overlapping genes. A Venn diagram depicting the number of overlapping genes with common and rare variants among the three families.

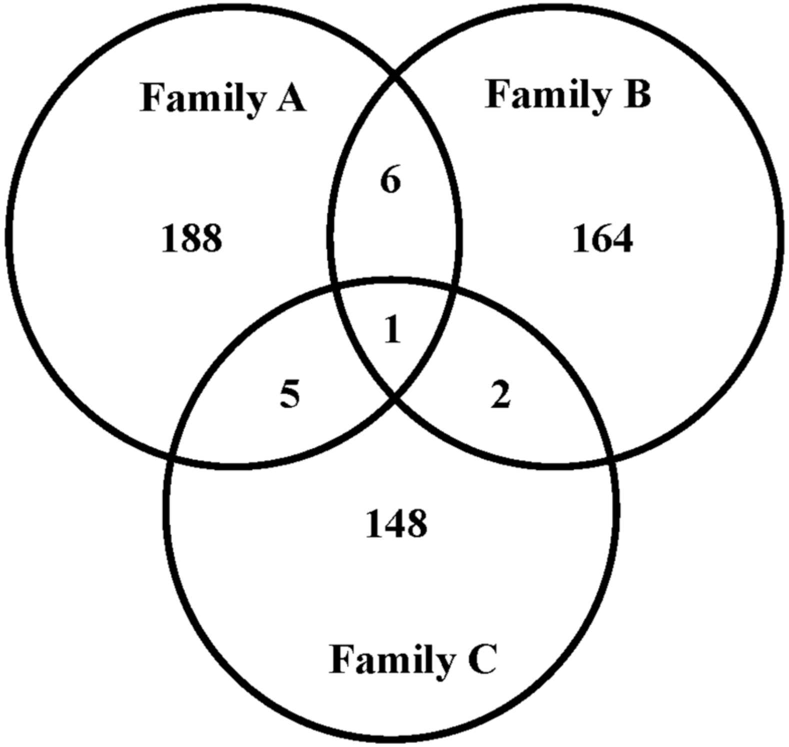

Supplement: Additional file 1: Table S1. — Clinical characteristics of the subjects. Table S2. Odds ratios (ORs) and 95% confidence intervals (CIs) for atopic dermatitis associated with COL6A6 polymorphisms in Korean patients. Table S3. Fliaggrin variants detected in three families. Figure S1. Pedigree diagrams of three families with atopic dermatitis. Figure S2. Flow chart of the process for filtering variants. Figure S3. A Venn diagram showing the number of overlapping genes. (PDF 258 kb) [file 12881_2017_368_MOESM1_ESM.pdf]
